# Impact of Seasonal, Environmental, and Inflammatory Factors on Chronic Urticaria Activity and Serum Biomarkers: A Prospective Cohort Study

**DOI:** 10.3390/jcm15020645

**Published:** 2026-01-13

**Authors:** Gulistan Alpagat, Ayşe Fusun Kalpaklioglu, Ayse Baccioglu

**Affiliations:** Department of Clinical Immunology and Allergic Diseases, Medical Faculty, Kirikkale University, Kirikkale 71450, Türkiye; afusunk@gmail.com (A.F.K.); aysebaccioglu@gmail.com (A.B.)

**Keywords:** air pollution, chronic urticaria, serum biomarkers, urticaria control test, weather conditions

## Abstract

**Background:** Chronic urticaria (CU) is characterized by recurrent wheals and/or angioedema persisting for more than six weeks. While disease triggers are often unidentified, seasonal and environmental factors may modulate disease activity; however, evidence regarding their clinical impact remains limited. **Objective:** This study aimed to evaluate the effects of seasonal, meteorological, and pollutant-specific environmental factors on urticaria control using the Urticaria Control Test (UCT), and to compare these effects between chronic spontaneous urticaria (CSU) and chronic inducible urticaria (CIU) in relation to inflammatory serum biomarkers. **Materials and Methods:** This prospective observational study was conducted at the Allergy and Clinical Immunology outpatient clinic of Kirikkale University Faculty of Medicine between 1 June 2023 and 1 April 2024. Patients with CU were classified as CSU or CIU according to international guidelines. Each participant was evaluated during summer and winter seasons. Area-level air pollution data and meteorological parameters were obtained from national monitoring systems. Disease control was assessed using the UCT, and inflammatory biomarkers were analyzed. **Results:** Urticaria control showed significant seasonal variation, with lower UCT scores during summer and higher scores during winter in both CSU and CIU patients. Among environmental factors, ozone (O_3_) was the only pollutant consistently associated with poorer urticaria control, whereas particulate matter and traffic-related pollutants, despite being higher in winter, showed no clinically relevant association. Summer months were characterized by increased inflammatory activity, including elevated leukocyte counts, neutrophil-to-lymphocyte ratio (NLR), C-reactive protein (CRP), and D-dimer levels, particularly in CSU patients. D-dimer emerged as an independent marker associated with poor disease control during summer. **Conclusions:** CU demonstrates marked seasonal variation, with disease worsening during summer months. Pollutant-specific effects, particularly O_3_ exposure, rather than overall air pollution burden, appear to be clinically relevant in urticaria control. Inflammatory and coagulation-related biomarkers may provide additional insight into disease activity. These findings support a season-aware and individualized management approach and highlight the need for future studies incorporating individual-level exposure assessment and biomarker-guided strategies.

## 1. Introduction

Urticaria is characterized by recurrent wheals, angioedema, or both, and is classified as acute or chronic according to symptom duration. Acute urticaria (AU) lasts less than six weeks, whereas chronic urticaria (CU) persists for six weeks or longer [1]. According to the EAACI/GA^2^LEN/EuroGuiDerm/APAAACI guideline, CU is further divided into chronic spontaneous urticaria (CSU) and chronic inducible urticaria (CIU), reflecting differences in triggering factors and underlying pathophysiology [2].

Although genetic and immunological mechanisms, including mast cell activation, play a central role in CU, external factors such as air pollution and weather conditions may modulate disease activity and symptom severity [3,4]. Environmental stressors can influence systemic inflammation and coagulation pathways, potentially contributing to fluctuations in urticaria control.

Air pollution consists of gaseous pollutants and particulate matter capable of inducing oxidative stress and inflammatory responses in exposed tissues [5]. Despite continuous skin exposure to these environmental factors, their clinical impact on CU control and potential differential effects on CSU and CIU remain insufficiently defined.

Few studies have simultaneously evaluated seasonal variations, environmental conditions, and inflammatory biomarkers in relation to CU activity [3,4]. Therefore, this prospective study aimed to investigate the effects of air pollution and meteorological factors on urticaria control, assessed using the urticaria control test (UCT), and to explore differences between CSU and CIU in relation to UCT scores and selected serum biomarkers.

## 2. Materials and Methods

### 2.1. Ethical Approval

All procedures involving human participants were conducted in accordance with the ethical standards of the institutional and/or national research committee and with the Declaration of Helsinki and its later amendments. Ethical approval for this study was obtained from the Kirikkale University Ethics Committee for Clinical Research (Approval Date: 29 September 2022; Decision No. 08/03). Written informed consent was obtained from all participants prior to enrollment.

### 2.2. Study Populations

This study was conducted as a prospective, observational, single-center study at the Allergy and Clinical Immunology Outpatient Clinic of Kirikkale University Faculty of Medicine with patient recruitment from 1 June 2023 to 1 April 2024. CU was defined as the presence of recurrent wheals, angioedema, or both persisting for at least six weeks. Patients with CU were classified as having CSU or CIU in accordance with the EAACI/GA^2^LEN/EuroGuiDerm/APAAACI international guideline. Classification was based on a detailed medical history, physical examination, and assessment of reproducible triggering factors. CSU was diagnosed in patients with recurrent wheals and/or angioedema occurring without an identifiable external trigger, whereas CIU was diagnosed when symptoms were consistently provoked by specific physical or environmental stimuli (e.g., pressure, cold, heat, or exercise). Participants were consecutively recruited from the Allergy and Clinical Immunology outpatient clinic during the study period. As this was a prospective observational study, no randomization or intervention was performed.

### 2.3. The Inclusion Criteria

(i) Diagnosed with CU by an allergy specialist; (ii) Aged >18 years, male or female; (iii) Provided informed consent to participate in the study; (iv) Residing in Kirikkale; (v) Receiving antihistamine treatment.

### 2.4. The Exclusion Criteria

(i) Declining to participate in the study; (ii) Residing outside Kırıkkale; (iii) Receiving biological therapy.

### 2.5. Urticaria Control Test (UCT)

The UCT is a validated patient-reported outcome measure consisting of four questions evaluating symptom severity, quality of life impairment, adequacy of treatment, and overall disease control during the preceding four weeks. Each item is scored from 0 to 4, yielding a total score ranging from 0 to 16. Urticaria control was defined as poor for scores < 12 and well controlled for scores ≥ 12 [6,7].

### 2.6. Environmental and Meteorological Data

Daily air pollution data, including particulate matter ≤ 10 µm (PM_10_), particulate matter ≤ 2.5 µm (PM2.5), nitrogen dioxide (NO_2_), sulfur dioxide (SO_2_), carbon monoxide (CO), and ozone (O_3_), were obtained from the Turkish Ministry of Environment and Urbanization monitoring network. Meteorological parameters, including temperature, relative humidity, rainfall, wind speed, and atmospheric pressure, were retrieved from the Turkish State Meteorological Service.

Seasonal mean values were calculated for summer and winter periods. Air pollutants were analyzed individually to allow pollutant-specific evaluation of their associations with urticaria control.

### 2.7. Laboratory Measurements

Blood samples were collected during scheduled visits and analyzed using standard laboratory methods at the central laboratory of Kırıkkale University Hospital. Laboratory assessments included complete blood count, C-reactive protein (CRP), and D-dimer levels.

### 2.8. Sample Size and Statistical Analysis

Statistical analyses were performed using SPSS software (version 21.0; IBM Corp., Armonk, NY, USA). Continuous variables were tested for normality and compared using Student’s *t*-test or the Mann–Whitney *U* test, as appropriate. Categorical variables were analyzed using the chi-square test. Associations between continuous variables were evaluated using Pearson’s correlation coefficient.

Multivariate logistic regression analysis was conducted to identify factors independently associated with poor urticaria control, and results are presented as odds ratios (ORs) with 95% confidence intervals (CIs). The UCT score was predefined as the primary outcome measure. As the analyses were hypothesis-driven and focused on predefined outcomes, no formal adjustment for multiple comparisons was applied; this approach has been explicitly stated to ensure transparency. A *p*-value < 0.05 was considered statistically significant.

## 3. Results

A total of 161 patients with CU were included in the study, including 83 patients with CSU and 78 patients with CIU. The demographic and clinical characteristics of the study population are presented in Table 1. Patients with CSU were significantly older and had a higher body mass index (BMI) than CIU patients, while sex distribution was similar between groups.

Seasonal meteorological conditions and air pollution parameters are summarized in Table 2. Summer was characterized by higher temperatures and lower humidity, whereas winter was colder and more humid. Concentrations of particulate matter (PM_10_ and PM_2.5_), NO_2_, SO_2_, and CO were higher during winter months. In contrast, O_3_ concentrations were higher during summer.

Seasonal comparisons of disease activity and key inflammatory markers are shown in Table 3. Both CSU and CIU patients demonstrated significantly poorer urticaria control during summer, reflected by lower UCT scores and a higher proportion of uncontrolled disease. Elevated D-dimer and CRP levels during summer were more pronounced in CSU patients.

Multivariate regression analysis identified D-dimer as the only independent factor significantly associated with poor urticaria control during summer (Table 4). No clinical, laboratory, or environmental parameters were independently associated with urticaria control during winter.

## 4. Discussion

The present study evaluated the impact of seasonal, meteorological, and environmental factors on disease control in CU, with a specific focus on pollutant-specific effects rather than overall air pollution burden. The principal finding of this prospective study is that CU exhibits clear seasonal variability, with significantly poorer disease control during the summer months. Importantly, O_3_ was identified as the only environmental pollutant consistently associated with worsening urticaria control, whereas other pollutants showed no clinically relevant effect.

Seasonal differences in disease activity were observed in both CSU and CIU, as reflected by lower UCT scores and a higher proportion of uncontrolled disease during summer. These findings are consistent with previous studies reporting increased urticaria-related symptoms and healthcare utilization during warmer periods [8,9,10,11,12]. Notably, despite higher concentrations of particulate matter and traffic-related gaseous pollutants during winter, disease control was better in this season, highlighting that the presence of air pollutants alone does not necessarily translate into clinical deterioration.

Among the evaluated environmental parameters, O_3_ emerged as the most relevant pollutant influencing urticaria activity. O_3_ concentrations were higher during summer and showed a negative association with urticaria control, whereas PM_10_, PM_2.5_, NO_2_, SO_2_, and CO predominated in winter when disease control was improved. O_3_ is a potent oxidant capable of disrupting the skin barrier, inducing oxidative stress, and activating pro-inflammatory signaling pathways in the skin [11]. These processes may enhance mast cell activation and amplify inflammatory responses, thereby facilitating wheal formation and symptom exacerbation. Our findings support the concept that pollutant-specific biological effects, rather than cumulative pollution exposure, are of greater clinical relevance in CU.

In addition to environmental influences, inflammatory and coagulation-related biomarkers demonstrated marked seasonal variation, particularly in CSU patients. During summer, CSU patients exhibited higher D-dimer and CRP levels, leukocytosis, neutrophilia, and elevated NLR, suggesting a more pronounced systemic inflammatory and pro-coagulant state compared with CIU patients, who showed relatively higher eosinophil counts. These subtype-specific inflammatory profiles are consistent with previous reports emphasizing heterogeneity in the immunopathogenesis of CSU and CIU [2,13,14,15].

Notably, D-dimer was identified as the only independent predictor of poor urticaria control during summer in multivariate regression analysis. This finding is in line with accumulating evidence linking activation of the coagulation cascade to CSU disease activity [16,17,18,19,20,21,22,23]. Coagulation pathway activation may promote thrombin generation, leading to increased endothelial permeability and mast cell activation, key mechanisms underlying wheal formation. As a fibrin degradation product, D-dimer may reflect ongoing endothelial activation and vascular leakage, both of which are central features of urticaria pathophysiology. The absence of significant predictors during winter further supports the notion that environmental and inflammatory triggers exert their greatest influence during warmer months.

From a clinical and translational perspective, these findings suggest that D-dimer may serve as a useful biomarker of disease activity, particularly in patients with poor control during periods of increased environmental stress. Although causality cannot be established due to the observational nature of the study, our results provide a rationale for future biomarker-driven research and raise the possibility that targeting coagulation–inflammation interactions could contribute to more personalized management strategies in selected CU patients.

The strengths of this study include its prospective design, seasonal evaluation, use of a validated disease control instrument (UCT), and integration of environmental and laboratory data. However, several limitations should be acknowledged. The study was conducted at a single center with a relatively limited sample size, environmental exposure was assessed at a regional rather than individual level, and causal relationships cannot be inferred. Furthermore, only summer and winter seasons were evaluated, and transitional seasonal periods were not assessed.

This study demonstrates that CU exhibits clear seasonal variation, with poorer disease control during summer months. O_3_ emerged as the environmental factor most closely associated with symptom exacerbation, whereas particulate matter and other traffic-related pollutants showed no clinically relevant effect. In addition, D-dimer was identified as a biomarker associated with poor urticaria control during periods of increased environmental stress, suggesting potential value in clinical monitoring. Elevated inflammatory markers, including neutrophil-to-lymphocyte ratio (NLR) and CRP, were also observed during periods of poorer disease control, indicating enhanced systemic inflammation. These findings support a pollutant-specific and season-aware approach to CU management and highlight the need for future studies incorporating individual-level exposure assessment and biomarker-guided strategies to advance personalized care in this patient population.

## Figures and Tables

**Table 1:** Demographic of patients with chronic urticaria.

Parameter	CSU (*n* = 83)	CIU (*n* = 78)	*p* Value
Age (years)	42.0 ± 16.5	33.9 ± 12.6	0.001
Female sex, *n* (%)	60 (72%)	54 (69%)	0.67
Body mass index (BMI) (kg/m^2^)	28.3 ± 5.9	25.8 ± 5.4	0.007

CSU, Chronic spontaneous urticaria; CIU, Chronic inducible urticaria.

**Table 2:** Seasonal meteorological conditions and air pollutant concentrations.

Parameter	Summer (Mean)	Winter (Mean)
Temperature (°C)	24.2	6.3
Relative humidity (%)	43.6	69.7
Atmospheric pressure (hPa)	925.8	931.6
PM_10_ (µg/m^3^)	40.4	47.1
PM_2.5_ (µg/m^3^)	5.1	20.7
NO_2_ (µg/m^3^)	22.5	37.1
SO_2_ (µg/m^3^)	19.5	29.7
CO (µg/m^3^)	343.7	760.5
O_3_ (µg/m^3^)	4.95	2.52

**Table 3:** Seasonal comparison of urticaria control and key inflammatory markers.

Parameter	CSU—Summer	CSU—Winter	CIU—Summer	CIU—Winter
UCT score	9.33 ± 2.93	11.64 ± 4.00	9.66 ± 3.17	11.47 ± 3.73
Uncontrolled urticaria (UCT < 12), %	77.5	23.5	74.3	28.8
D-dimer > 500 ng/mL, %	57.3	25.5	39.7	21.7
CRP > 5 mg/L, %	31.7	28.2	14.1	19.6
NLR	2.78 ± 0.33	2.13 ± 0.89	2.46 ± 1.61	2.26 ± 1.13

**Table 4:** Multivariate regression analysis of factors associated with urticaria control during summer.

Variable	Odds Ratio	95% CI	*p* Value
D-dimer (mg/L)	1.001	1.000–1.003	0.040
Age (Years)	0.99	0.95–1.04	0.67
Sex (Female)	0.15	0.02–1.07	0.058

## Data Availability

The data that supports the findings of this study are available on request from the corresponding author. The data is not publicly available due to privacy or ethical restrictions.
